# Gut microbiome is affected by inter-sexual and inter-seasonal variation in diet for thick-billed murres (*Uria lomvia*)

**DOI:** 10.1038/s41598-020-80557-x

**Published:** 2021-01-13

**Authors:** Esteban Góngora, Kyle H. Elliott, Lyle Whyte

**Affiliations:** Department of Natural Resource Sciences, McGill University, 21111 Lakeshore Road, Sainte-Anne-de-Bellevue, H9X 3V9 Canada

**Keywords:** Bioinformatics, High-throughput screening, Microbial ecology, Stable isotope analysis, Microbial communities, Behavioural ecology

## Abstract

The role of the gut microbiome is increasingly being recognized by health scientists and veterinarians, yet its role in wild animals remains understudied. Variations in the gut microbiome could be the result of differential diets among individuals, such as variation between sexes, across seasons, or across reproductive stages. We evaluated the hypothesis that diet alters the avian gut microbiome using stable isotope analysis (SIA) and 16S rRNA gene sequencing. We present the first description of the thick-billed murre (*Uria lomvia*) fecal microbiome. The murre microbiome was dominated by bacteria from the genus *Catellicoccus*, ubiquitous in the guts of many seabirds. Microbiome variation was explained by murre diet in terms of proportion of littoral carbon, trophic position, and sulfur isotopes, especially for the classes Actinobacteria, Bacilli, Bacteroidia, Clostridia, Alphaproteobacteria, and Gammaproteobacteria. We also observed differences in the abundance of bacterial genera such as *Catellicoccus* and *Cetobacterium* between sexes and reproductive stages. These results are in accordance with behavioural observations of changes in diet between sexes and across the reproductive season. We concluded that the observed variation in the gut microbiome may be caused by individual prey specialization and may also be reinforced by sexual and reproductive stage differences in diet.

## Introduction

The role of bacteria in higher trophic level predator interactions is increasingly being appreciated^1,2^. The gut microbiome may be crucial for the adaptation of the vertebrate host to ecological pressures, such as rapid environmental change or adverse conditions^3–6^. Bacterial communities play an important role in processing food, and matching microbiome with diet may be one mechanism for animals to overcome changes in their diet throughout their life cycle^7,8^. Variation in diet composition was linked to changes in the gut microbiome in wild sticklebacks and perches, two fish species with individual prey specialization (IPS)^7,8^. IPS is a widely distributed phenomenon, occurring in a large number of taxa where individuals of the same population use prey resources differentially^9,10^. In particular, individuality in prey selection is not explained by obvious differences, such as by sex or age^9,10^. These studies also show that other inter-individual variables can change the feeding behaviour (e.g. differential diets between sexes) leading to variation in the microbiome^6,8^.

Most studies describing diet effects on the avian gut microbiome are based on domestic or captive birds such as chickens^11–13^ or raptors^14^. These studies have mainly focused on the detection of specific groups or species of bacteria (mostly pathogens)^15–17^. Recently, gut microbial communities for a few wild bird species have been described^18^. Maul and collaborators^19^ characterized cloacal bacteria according to carbon substrate utilization in culture plates (EcoPlates) and clustered these bacterial communities of various passerine bird species according to bird diet. The gut microbiome of two passerine species differed between spring and fall migrations^6^ and between migrating and resident shorebirds^20^, suggesting that the environment, and possibly diet, has a strong influence on wild bird microbiomes. Dewar et al*.*^21^ reported individual variation in the gastrointestinal microbiota of four penguin species. One meta-analysis illustrated that, even among phylogenetically and behaviourally diverse species, diet has an important effect on gut microbiome composition^22^. Lower microbial diversity of house sparrow guts from urbanized areas compared to rural sparrows shed some light to the way in which microbiomes can adapt to reduced niches (a city, compared to the countryside), but may also lose plasticity^23^. Hird and collaborators^24^ studied the intestinal microbiome of 59 Neotropical bird species and determined that host species was the most important determinant for the intestinal microbial composition, followed by host ecology (i.e. broad dietary preferences and habitat)^24^. They also proposed the inclusion of gut sampling as part of the field sampling protocol for collection of museum specimens as it could provide valuable information to the “microdiversity” within wild macroorganisms^24^. Similar results were obtained for the gut microbiomes of 74 Equatorial Guinean bird species in terms of the importance of host species, diet, and location as determinants of the microbial composition^25^. The gut microbiomes of Darwin’s finches were shown to be conserved across species with the exception of the blood-feeding vampire finch, showing that extreme diet specialization can change microbial gut composition^26^.

Thick-billed murres (*Uria lomvia*; hereafter ‘murres’) are the most abundant seabirds in the Canadian Arctic. In the low Arctic, murres are generalists, with the most diverse diet of any *Uria* population. However, despite being population-wide generalists, there is a high degree of IPS that is maintained across time with some individuals disproportionately catching rare prey types year after year^27,28^. Sex-specific feeding behaviours are also present as males tend to feed on amphipods and shallow benthic prey at night, while females tend to feed on deep benthic and schooling prey only available during the day^29,30^. In addition, females feed at a higher trophic level when rearing chicks compared to when they are incubating their egg, but males do not^29^.

In this study we used 16S rRNA gene sequencing of fecal samples as a proxy to describe for the first time the gut microbiome of the thick-billed murre. We tested the hypothesis that diet alters gut microbiome. For this, we used stable isotope ratios as direct estimators of diet. As ^15^N content in an organism increases systematically as trophic level increases, the nitrogen isotope ratio (δ^15^N) is useful for describing trophic position^31^. The carbon isotope ratio (δ^13^C), also referred to as the proportional use of littoral carbon^8^, can help to determine diet according to the habitat animals feed on as, for example, there is a systematic enrichment of ^13^C in benthic feeding organisms, relative to pelagic feeding organisms^32^. Sulfur stable isotopes present can also be used to determine diet through changes in habitat as the most-enriched δ^34^S values are found in the benthos of marine systems^33^. We also examined variation in the murre gut microbiome between sex and reproductive stage (incubation vs. chick-rearing), that would be consistent with known variations in murre diet between sexes and reproductive stages^29^.

## Results

### The composition of the murre fecal microbiome is dominated by the genus *Catellicoccus*

**Figure 1:**
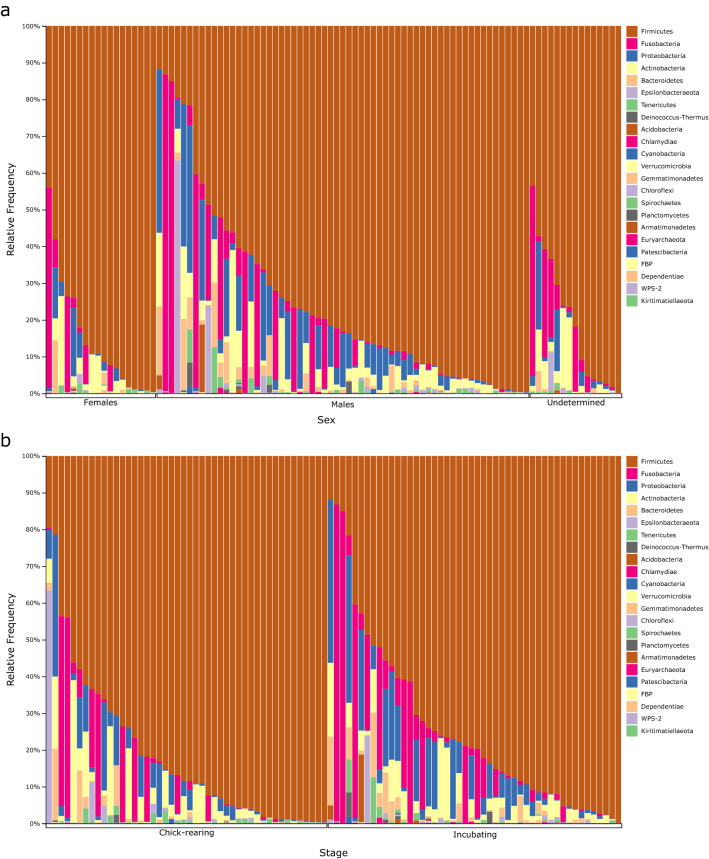
Taxonomic classification at the Phylum level of the ASVs obtained from murre fecal samples showing a strong influence of bacteria belonging to the Phylum Firmicutes. Samples were grouped by (**a**) sex of the sampled bird or (**b**) the reproductive stage at which the bird was during sampling.

**Figure 2:**
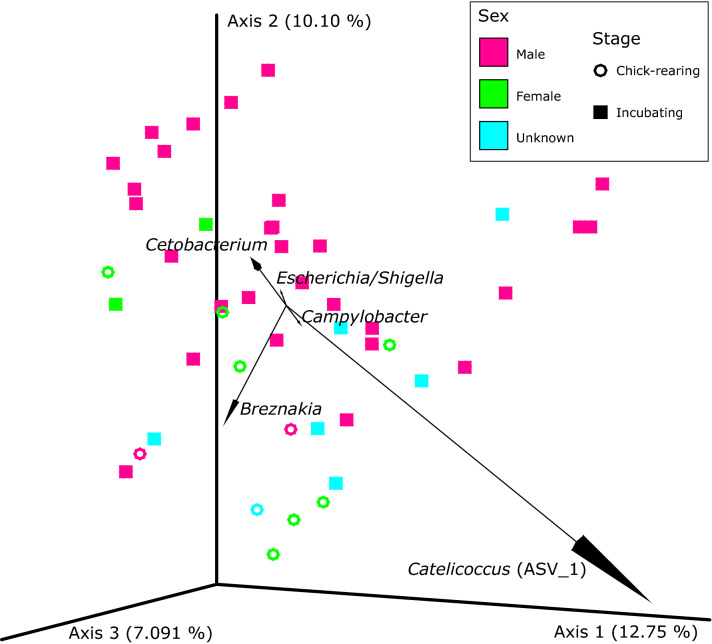
Principal Coordinates Analysis (PCoA) plot for the gut community composition using the unweighted UniFrac metric with samples identified by sex and reproductive stage. Biplots show the taxonomy of the ASVs with the top 5 effects on the community composition with position of the arrow indicating the direction of the effect.

After denoising and filtering, a total of 7,232,617 16S rRNA gene reads with an average of 76,942 ± 4960 (mean ± SE; n = 94) reads per sample were obtained. We observed a large degree of inter-individual variation in the composition of the gut microbiome within the studied individuals (Fig. 1). Firmicutes was the predominant phylum and ranged from over 11.9% to over 99.8% of the microbiome of the sampled murres with *Catellicoccus* being the dominant genus detected and comprising 1.6% to 98.8% of an individual’s gut microbiota (Supplementary Figs. 1 and 2). Other major phyla in the murre gut microbiome include Fusobacteria, Proteobacteria, and Actinobacteria. There is a strong influence of some amplicon sequence variants (ASVs) on the composition of the murre gut microbiome (Fig. 2). The most important features determining the weighted and unweighted UniFrac distance matrix showed the influence of ASVs from the genera *Catellicoccus*, *Breznakia*, *Cetobacterium*, *Escherichia*/*Shigella* (these two genera cannot not be easily distinguished solely based on 16S rRNA gene sequences), and *Campylobacter* on the overall community composition of the murre fecal microbiome (Fig. 2 and Supplementary Fig. 3).

### Diet variation measured between sexes and reproductive stages is associated with individual ASV relative abundances

**Figure 3:**
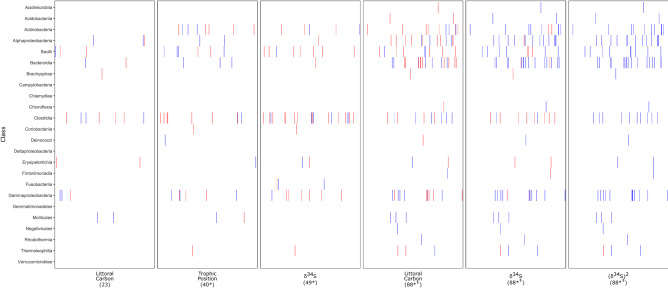
Quasibinomial effects of various diet metrics on the abundance of the 424 most abundant ASVs of the murre fecal microbiome. Columns contain the models of a diet metric for ASVs from a bacterial class in each row. Vertical bars represent an ASV within the given class that accounts for > 0.01% mean relative abundance and that has a positive or negative association with a particular metric. For ASVs with a red bar, its relative abundance increases with the metric. For ASVs with a blue bar, relative abundance decreased with the metric. Numbers under the different diet metrics indicate the number of ASVs for which an effect of the metric was observed. Numbers with an asterisk (*) indicate that the proportion of ASVs with an effect of the metric surpasses the expected 5% false positive rate. Metrics with a double dagger (‡) form part of the same model which combined the linear effect of the proportion of littoral carbon and the quadratic effect of δ34S.

Females fed at a higher trophic position and had larger δ^34^S values compared to males (Table 1). Individuals of both sexes fed at a higher trophic position during the chick-rearing stage than during the incubation stage (Table 1). Quasibinomial generalized linear models (GLMs) showed that 20.8% of the relative abundances of the most common (> 0.01% relative abundance) ASVs are correlated with a combination of the proportional use of littoral carbon and the quadratic effect of δ^34^S (Table 2). A χ^2^ test showed that this proportion statistically differs from chance with the expected 5% false detection rate (FDR) expected from performing multiple comparisons. For this group of models, ASV abundance tended to be mostly positively associated with the proportional use of littoral carbon and negatively associated with the quadratic effect of δ^34^S (Fig. 3). After performing an FDR correction, 19.6% of the models showed a correlation. Trophic position and δ^34^S also correlated with ASV relative abundance at a larger proportion than the 5% FDR (Fig. 3; Table 2). After the FDR correction, 1.2% of the ASVs were correlated with the proportional use of littoral carbon, 1.4% were correlated with trophic position, and 0.5% with δ^34^S.

**Table 1:** Comparison of stable isotope values between sexes and reproductive stage.

	Sex	Mean	SD	*t*	*p*
Trophic position	Female	3.79	0.11	7.10	4.53 × 10^–5^
	Male	3.48	0.09		

	Sex	Mean	SD	*W*	*p*
Proportional use of littoral carbon	Female	0.42	0.05	141	0.46
	Male	0.41	0.04		
δ^34^S	Female	18.86	1.24	177.5	0.04
	Male	18.06	0.56		

	Stage	Mean	SD	*W*	*p*
Trophic position	Chick-rearing	3.75	0.18	259	9.03 × 10^–4^
	Incubating	3.51	0.10		
Proportional use of littoral carbon	Chick-rearing	0.41	0.05	151	0.96
	Incubating	0.42	0.04		
δ^34^S	Chick-rearing	18.7	1.28	206.5	0.11
	Incubating	18.1	0.58

**Table 2:** Proportion of quasibinomial GLMs explaining the relationship of various isotope signatures with ASV abundance.

Isotope signature	Percentage of models explaining ASV abundance	*p*_*χ*2_	Percentage of models explaining ASV abundance after FDR
*alpha*	5.4	0.69	1.2
Trophic position	9.4	2.80 × 10^–5^	1.4
δ^34^S	11.6	5.84 × 10^–10^	0.5
*alpha* + (δ^34^S)^2^	20.6	4.13 × 10^–50^	19.6

*alpha* = proportional use of littoral carbon.

### Microbial community metrics differ between reproductive stages and sexes

**Figure 4:**
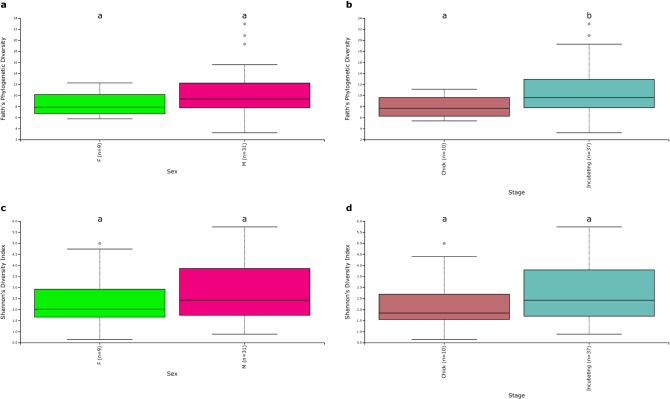
Differences in Faith’s phylogenetic diversity for the gut microbiome with no differences between sexes (**a**) but evidencing differences between reproductive stages (**b**) and showing no differences in terms of Shannon’s Diversity Index (**c**) between sexes or (**d**) between reproductive stages. Letters above boxplots represent differences between groups with p < 0.05.

**Figure 5:**
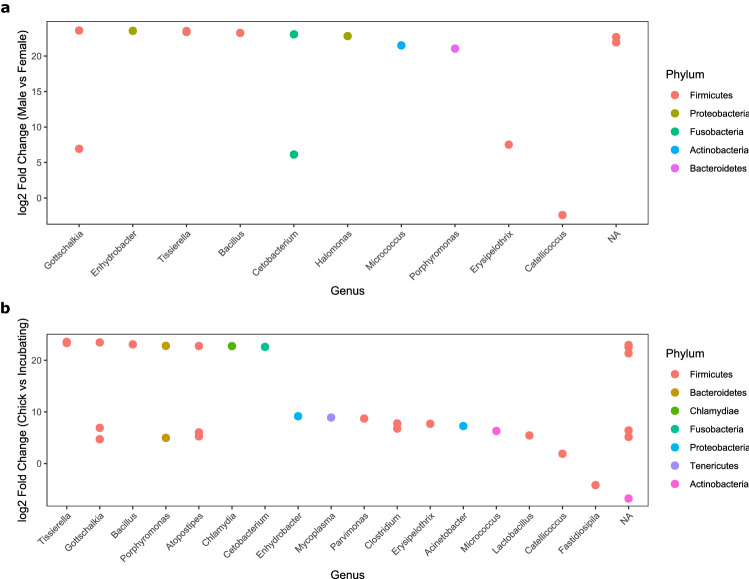
Differences in abundance of various ASVs in the murre microbiome between sexes (**a**) and reproductive stages (**b**). Each point represents an ASV from a particular class that is more or less abundant in one group than the other. Colors represent the different phyla these ASVs belong to.

No differences in Faith’s phylogenetic diversity (Faith’s PD) were found between sexes (Fig. 4a; Table 3). Incubating birds had a more phylogenetically diverse microbiome than chick rearing birds (Fig. 4b; Table 3). No differences were observed between sexes or reproductive stages in terms of Shannon’s diversity index (Fig. 4c,d; Table 3). Murres appeared to have different microbial communities between reproductive stages in terms of qualitative community composition (Fig. 2a; Supplementary Fig. 4a): unweighted UniFrac distances (PERMANOVA: pseudo-*F* = 1.99, R^2^ = 0.042, *P* = 0.007 ). No differences for unweighted UniFrac distances were observed between sexes. It must be noted that the multivariate dispersions are not homogeneous between incubating and chick-rearing birds (PERMDISP: *F* = 9.89, *P* = 0.015) with greater dispersion among incubating birds than among chick-rearing birds. There were no statistical differences between the dispersions of males and females. No differences were observed between sexes or reproductive stages for the weighted UniFrac distances (Supplementary Figs. 3 and 4b). We also observed different abundances of individual ASVs between sexes as 34 ASVs were more abundant in males than in females (Fig. 5a; Supplementary Table 1). There were 13 ASVs which were more abundant for birds during the chick-rearing stage and 24 ASVs that were more abundant for birds during the incubation stage (Fig. 5b; Supplementary Table 1).

**Table 3:** Kruskal–Wallis tests for differences in Faith’s phylogenetic diversity and Shannon’s diversity index between sexes and reproductive stages.

	Sex	Mean	SD	*H*	*p*
Faith's PD	Female	8.34	2.32	1.99	0.159
	Male	10.53	4.40		
Shannon's diversity	Female	2.49	1.48	0.44	0.507
	Male	2.76	1.23		

	Stage	Mean	SD	*H*	*p*
Faith's PD	Chick-rearing	7.96	2.13	3.90	0.048
	Incubating	10.74	4.34		
Shannon's diversity	Chick-rearing	2.32	1.38	0.88	0.349
	Incubating	2.72	1.24

## Discussion

As expected, diet—as measured by stable isotope analysis (SIA)—was associated with variation in gut microbiome. The association was linear for trophic position and carbon source but was non-linear for sulfur source. We observed large amounts of individual-level variation that could be caused by diet specialization observed previously for this species^27–29^. Moreover, microbiome changed with sex and reproductive stage, which is also consistent with known dietary differences.

The gut microbiome of the thick-billed murre is consistent with other avian microbiomes, which are mainly comprised of the phyla Firmicutes, Actinobacteria, Bacteroidetes, and Proteobacteria^18^. Even though ethanol preservation can lead to changes in the relative abundance of some bacterial groups, these changes are smaller or comparable to observed differences among technical replicates^34^. Because of this, we do not consider that the patterns in our study showing the dominance of Firmicutes to be an artifact of the preservation method. However, we did observe that there was only a small proportion of Bacteroidetes in our samples compared to other studies^18^ (Fig. 1). Bacteroidetes have been associated with degradation of complex biopolymers, such as cellulose, in mammal guts and it has been suggested that this association may also exist for birds^35^. Given that plant material is not a regular part of the murre diet, this could explain the low abundance of Bacteroidetes in our study.

The murre gut microbiome was dominated by bacteria belonging to the genus *Catellicoccus* (phylum Firmicutes). This genus was first described with isolates from harbour porpoise (*Phocoena phocoena*) and grey seal (*Halichoerius grypus*) carcasses^36^ and, since then, have been found to be ubiquitous in the gut microbiome of various avian species. *Catellicoccus marimammalium* is used as a marker for detection of gull fecal contamination^37–40^. Bacteria from the genus *Catellicoccus* have also been found in the gastrointestinal tract of zebra finches (*Taeniopygia guttata*)^41^; barn swallows (*Hirundo rustica*)^42^; red knots (*Calidris canutus*) and ruddy turnstones (*Arenaria interpres*)^43^; black-tailed godwits (*Limosa limosa*), black-winged stilts (*Himantopus himantopus*) and common redshanks (*Tringa totanus*)^44^; black-headed gulls (*Chroicocephalus ridibundus*)^45^ and waterfowl^46^. The genome of *Catellicoccus marimammalium* revealed that this bacterium encodes various functions such as nutrient transport and bile acid hydrolysis suggesting a symbiotic lifestyle of this species^46^. Possible beneficial effects for the host such as immune modulation and gut maturation have also been proposed for bacteria of this genus inhabiting the avian gut^41^. Additionally, members of the Firmicutes are widely involved in the production of short-chain fatty acids which can be absorbed as an energy source by the host gut wall^35^. Firmicutes have also been associated with weight gain, increase of nutrient uptake and metabolic efficiency in chickens^35^. It is then possible to hypothesize that *Catellicoccus* detected in the murre gut are aiding these birds to optimize their nutrition as the murres face the harsh conditions of the Arctic and the limited feeding events during the reproductive season.

High abundances of bacteria from the phylum Fusobacteria have also been observed in the guts of other seabirds such as gentoo and king penguins (*Pygoscelis papua* and *Aptenodytes patagonicus*, respectively)^21,47^, common diving petrels (*Pelecanoides urinatrix)*^48^, gulls^40^, vultures and carnivorous mammals^18,49^, and humans^50^. Although bacteria from this phylum are known to be pathogenic, recently it has been observed that they could aid their host to metabolize nutrients^18,49^. Fusobacteria are known butyrate producers and can ferment amino acids and glucose^47,48,50^ and, in chickens, they boost the host immune system and adiposity^48^. The most influential genus observed for this phylum that shaped the overall community composition (Fig. 2) was *Cetobacterium*. *Cetobacterium someare* isolated from the intestinal tract of freshwater fish produce vitamin B_12_ and acetic acid which suggest the beneficial effects of this bacterium for its host^51^.

The biplot analysis for the UniFrac PCoA plots (Fig. 2 and Supplementary Fig. 3) showed that one of the most influential ASVs shaping the microbial community structure belonged to the genus *Breznakia*. This genus is relatively novel and not much is known about these bacteria, but this genus and others in the family Erysipelotrichaceae have been frequently isolated from the guts of mammals and insects, making this family mostly comprised of inhabitants of animal intestinal tracts^52^. Many bacterial genera associated with opportunistic pathogens, such as *Campylobacter*, *Helicobacter*, *Escherichia*/*Shigella*, *Corynebacterium*, *Mycobacterium*, *Neisseria* and *Ornithobacterium*, among others, were found in the murre gut. However, no risk of disease is believed to exist solely based of the presence of these genera as many of them contain representatives that have also been found in other healthy birds^21,43,44,47,53^. The presence of these taxa should continue to be monitored given that reverse zoonosis of various *Campylobacter* strains associated with humans occurs in Antarctic seabirds^54^.

The gut microbiome of males and females are different in terms of qualitative community composition (Fig. 2) and abundances of individual ASVs (Fig. 5a). The SIA also showed that females are feeding at a higher trophic position than males, suggesting that trophic position influences the fecal microbiome of murres. These results are consistent with the social behaviour of murre mating pairs at Coats Island in which males stay in the colony during the day and leave their nest to feed at night while females feed during the day and go back to the colony during the night^29^. This leads to different types of prey being caught by each sex, with males preferring amphipods and shallow diving fish and females preferring deep water benthic prey^29^. These observations are also consistent with the differences we detected in terms of δ^34^S as females had a larger average value for this isotopic signature, which suggests that they are feeding at higher depths than males. Additionally, risk-prone diet for females (i.e. schooling fish)^29^ could provide less consistent food sources which helps to explain why there was a smaller phylogenetic diversity for females and that some key genera, such as *Cetobacterium*, were more abundant in males.

We detected differences in phylogenetic diversity (Fig. 4b) and in qualitative composition of bacterial communities between reproductive stages (Fig. 2). We also observed individual ASVs that changed in abundance between the chick-rearing and incubating birds (Fig. 5b). This may occur because, for example, chick-rearing females feed at higher trophic levels than incubating females, as chick-rearing females must locate fish to bring back and feed their offspring^29^. SIA confirmed that chick-rearing murres were in fact feeding at a higher tropic level than those sampled during the incubation stage. Diet variation caused by changes in prey composition throughout the breeding season could potentially cause the differences in the gut microbiome that we observe. As prey from higher trophic levels, such as fish, tend to be encountered less frequently^29^, dietary intake can become less consistent which could in turn modify the gut microbiome. Despite the fact that we detected heterogeneity of multivariate dispersions between reproductive stages for the unweighted UniFrac distances, PERMANOVA results have been observed to become too conservative for unbalanced designs when there is a greater dispersion for the largest group, as is the present case^55^. This suggests that the observed differences might in fact be larger than those detected by the PERMANOVA analysis between reproductive stages. Additionally, the lack of observed differences for the weighted UniFrac distances between sexes and reproductive stages suggest that the differences that we observe are caused due to differences in microbial richness, rather than in abundance in terms of the overall community composition.

However, we did observe some differences in individual ASVs’ abundance that cannot be explained by trophic position, sex, or reproductive stage but that can still be attributed to diet. The abundances of a large proportion of the most abundant ASVs were associated with the proportional use of littoral carbon and the quadratic effect of sulfate availability (δ^34^S). A differential use of littoral carbon suggests that an individual’s feeding preference on benthic or littoral food sources changes the abundance of certain groups of bacteria. δ^34^S is an isotope signature that has been used to differentiate between coastal and marine feeding birds^56^ and between sources of sulfate originated from the surface and the sediments^33^. Given that we observed both positive and negative quadratic effect of this isotope ratio, certain microbes appear to benefit from a more generalized diet and that some others benefit more from more specialized diets. Given the larger proportion of GLMs including the source of the sulfate and the use of littoral carbon compared to the GLMs using trophic position, the source of the food in terms of depth is likely a more important factor in shaping the microbiome than the trophic position of the prey that the birds feed on. These results support the behavioural observations of IPS at the murre colony in Coats Island^28^.

We conclude that the changing diet of the thick-billed murre between sexes and through the reproductive stage is linked to variation of the gut microbial communities. However, the correlational associations observed in the present study cannot be used to establish a causal effect of dietary preferences shaping the gut microbiome. There is an additional need for studies of wild animal microbiomes given that the behaviours and environmental conditions that might be encountered in nature could result in changes in the gut microbiome that might not be observable under laboratory conditions. For example, our study design was biased towards males (because they are more easily sampled at the colony), and future studies could focus on species that can be sexed morphologically allowing direct incorporation of sex into study design and capture protocol. We also corroborate the need to characterize diet for particular individuals instead of generalizing diets of populations as a whole^9,10^. Future studies could also focus on linking the trends in the composition of the microbiome that we describe with the fitness of individuals to determine how certain groups of bacteria are positively or negatively affecting their host birds.

## Methods

### Fecal sample collection and preparation

Methodologies were approved by the Macdonald Campus Facility Animal Care Committee under Animal Use Protocol number 2015–7599 and adhered to all regulations and guidelines. Thick-billed murres (N = 48; 9 females, 31 males, and 8 with undetermined sex) were captured with a noose pole and sampled between July and August 2017 at a breeding colony at Coats Island (62°98′N, 82°00′W) located in northern Hudson Bay, Nunavut, Canada. Fecal samples were aseptically taken by inserting sterile swabs into the cloaca. The swab was swirled inside the cloaca to stimulate the release of feces. The recovered fecal matter was collected in sterile polypropylene tubes which were kept on ice until they were taken back to the base camp (less than an hour from the moment of sampling). Samples were then mixed with absolute ethanol (4:1 ethanol to feces ratio) and stored at − 20 °C until processed (between 2 and 3 months after they were frozen). This process was done at two different time points with 2–7 days in between each sampling event (Supplementary Table 2)^57^. Ethanol was removed from the samples by centrifugation: polypropylene tubes containing the samples were centrifuged at 4500 RPM and 4 °C for 5 min and the supernatant was discarded. The pellet was resuspended in 2 mL of sterile deionized water, centrifuged at 4500 RPM and 4 °C for 5 min and the supernatant was discarded once more to assure any remaining ethanol was removed from the collected feces.

### DNA extraction and sequencing

Due to the high content of contaminants present in avian feces (e.g. uric acid) which might inhibit DNA extractions^58^, we modified the protocols of commercially used DNA extraction and purification kits to improve the quality of the obtained nucleic acids. DNA was extracted from the resulting pellets of the fecal samples using the DNeasy PowerLyzer PowerSoil kit (Qiagen, Hilden, Germany) according to the manufacturer’s instructions with minor modifications: samples were heated at 65 °C for 10 min before bead beating, and, for the final elution step, nuclease-free water was heated to 70 °C and used to elute the DNA. Extracted DNA was purified using Monarch PCR & DNA Cleanup Kit (New England Biolabs, Ipswitch, MA) according to the manufacturer’s instructions with minor modifications: 20 µL of nuclease-free water heated to 70 °C were used to elute the DNA.

The 16S rRNA gene was amplified using primers 515F-Y (5′-GTGYCAGCMGCCGCGGTAA) and 926R (5′-CCGYCAATTYMTTTRAGTTT)^59^ containing Illumina overhang adapter sequences. These primers were selected as they have been shown to be more accurate and produce longer amplicons than the 515F-C/806R primer pair, helping to differentiate previously unresolvable taxa^59^. 25 µL PCR reactions containing 1X HotStarTaq Master Mix (Qiagen, Hilden, Germany), 0.6 µM of each primer, 0.4 mg mL^−1^ BSA (Sigma-Aldrich, St. Louis, MO), and 1 µL of DNA were performed under the following conditions: initial denaturation at 95 °C for 15 min, followed by 25 to 35 cycles (depending on the DNA concentration of the sample) of 94 °C for 1 min, 50 °C for 45 s, 72 °C for 1 min, and a final extension step at 72 °C for 10 min. Reactions were purified using Agencourt AMPure XP magnetic beads (0.8 bead-to-PCR volume ratio; Beckman Coulter, USA). Indexing was performed using the Nextera XT index kit (Illumina, San Diego, CA) following manufacturer’s instructions with a minor modification of 15 min at 95 °C in the initial denaturation used to activate the polymerase (Qiagen HotStarTaq Master Mix). Indexed samples were purified with AMPure XP beads (1.12 bead-to-PCR volume ratio) and quantified using the Qubit fluorometer (Invitrogen, Thermo Fisher Scientific, USA). Samples (including negative controls of PCR reactions) were pooled in equimolar ratios of 4 nM and sequenced with a 2 × 250 bp run with v2 chemistry on a MiSeq platform (Illumina, San Diego, CA). Adapters and indices were removed by the Illumina FASTQ file generation pipeline.

### Sequencing data processing

Unless stated otherwise, all analyses were performed in R 3.6.1. Chimeras were removed from the raw sequencing data and a preliminary taxonomic assignment was undertaken with the Silva database^60^ version 132 release and using DADA2^61^. Sequencing data was then analyzed using Qiime 2^62^ (release 2020.8). Based on the preliminary classification, ASVs predominantly present in the negative controls (50% prevalence threshold) and also present in the fecal sample data were removed (n = 114), samples with fewer than 1000 sequences (n = 1), singletons (n = 70), and the negative controls (Supplementary Figure 7) were removed from further analysis. A Taxonomic classification was performed with the q2-feature-classifier multinomial naive Bayes classifier^63^ using the SSU Ref NR 99% Silva database^60^ version 132 release. ASVs that were not assigned a taxonomic classification at the domain level (n = 33), and mitochondrial and chloroplastic ASVs (n = 34), were removed. A phylogenetic tree was inferred using the approximately maximum-likelihood method implemented by FastTree 2^64^.

### Stable isotope analysis (SIA)

Blood was taken from the brachial vein using heparinized syringes. Red blood cells (RBCs) were separated from plasma by centrifugation and they were frozen and transported in gaseous nitrogen to the processing facility where they were stored at − 80 °C until processed. We followed the methodology previously used to perform SIA for murre blood samples from the same colony^2^. We freeze-dried and powdered the RBCs and lipids were removed from the powder using a 2:1 chloroform:methanol soak and rinse^65^. Stable isotope analyses for nitrogen (trophic position), carbon (proportional use of littoral carbon), and sulfur (feeding habitat depth) stable isotopes were performed by the G.G Hatch Stable Isotope Laboratory (Ottawa, ON) for 43 of the sampled birds that where also part of the Northern Contaminants Project of Aboriginal Affairs and Northern Development Canada. 1 mg subsamples were used for stable nitrogen and carbon isotope assays. All measurements are reported in parts per thousand (‰) relative to the AIR international standard. For every 10 samples, replicate measurements of internal laboratory standards calibrated to international standards were also run indicating a measurement error of ± 0.2‰. 10 mg subsamples were used to measure stable sulfur isotope ratios. All measurements are reported in parts per thousand (‰) relative to the VDCT international standard. Data was normalized with a precision of ± 0.4‰ using calibrated internal standards. Sample duplicates for every 10 samples were used for quality control to assure that the relative percent difference is less than 5%.

We used the δ^15^N and δ^13^C values obtained from the murre blood samples along with those of pelagic and littoral prey items commonly captured by murres^2^ in order to determine proportional use of littoral carbon and trophic position^8^ in R 3.6.1.

### Statistical analyses

Alpha level for all tests was 0.05. Unless stated otherwise, all analyses were performed in R 3.6.1. ASV abundances were converted into relative abundances for sequencing data normalization^66^. Repeatability was evaluated for birds where two samples were obtained using the q2-longitudinal plugin^67^ to compare differences in terms of alpha diversity with the Wilcoxon signed-rank test^68^. We also evaluated repeatability for the community composition between the two time points with a PERMANOVA test^69^ for unweighted and weighted UniFrac distances^70^. These repeatability analyses showed that samples from the first time point were not statistically different from samples for the second time point (Supplementary Figs. 5 and 6). Given these results, only the sequencing data from the first sampling point will be analyzed to account for the fact that the two sample points are not statistically independent.

Diversity analyses were performed with the q2-diversity plugin. Faith’s PD^71^ and Shannon’s diversity index^72^ were calculated for each sex and reproductive stage (incubating vs. chick-rearing). Kruskal–Wallis rank tests^73^ were used to observe statistical differences in Faith’s PD and Shannon’s diversity index between these groups. Unweighted and weighted UniFrac distances^70^ were calculated to observe the community structure of the murre gut microbiome. After testing for the homogeneity of multivariate dispersions (PERMDISP)^74^, differences in community structure for the two distance measurements between sexes and between reproductive stages were calculated using the permutational multivariate analysis of variance (PERMANOVA)^69^. Principal Coordinates Analysis (PCoA) plots and biplots^75^ were created with Emperor^76,77^ to visualize the differences obtained with PERMANOVA. Additionally, NMDS plots were created with phyloseq^78^ to provide a different visualization of the community composition.

We used DESeq2^79,80^ to observe differences in ASV abundance between sexes and reproductive stages. Given that this approach already corrects for differences in sample counts^80^, the differential expression analysis using a negative binomial distribution was applied on pre-normalisation by proportions data set as implemented by the DESeq function of the DESeq2 package^79^.

We tested differences of trophic position between sexes using Student’s t-test and we tested differences of isotopic signatures between sexes and reproductive stages with a Mann–Whitney test^81^, given the non-parametric nature of the isotope data for these groups. We also observed the effect that isotopic signatures had on the relative abundance the ASVs in our samples following a similar strategy as developed by Bolnick and collaborators^7,8^. We selected the most abundant ASVs that had a mean relative abundance of > 0.01% obtaining a total of 926 ASVs. We then used GLMs with a quasibinomial error created with the MuMIn package^82^ to evaluate the dependency of the abundance of individual ASVs on proportional use of littoral carbon, trophic position, and δ^34^S. Additionally, we evaluated if there were non-linear effects on ASV abundance using quadratic terms for the three isotopic signatures. To account for false positives, we used a chi-squared test to identify whether the number of GLMs with *p* < 0.05 exceeded the 5% null expectation. We then applied an FDR analysis to obtain the number of models for which *q* < 0.05.

## Supplementary Information

Supplementary Information.

## Data Availability

The sequencing data was deposited in Sequence Read Archive of NCBI under accession number PRJNA594033. Isotope data and sex data is included as the Supplementary Table 2 and it also available in the Mendeley Data repository, http://dx.doi.org/10.17632/r69xp7xsky.3^57^. Detailed parameters for the bioinformatic and statistical analyses used for the study is available in the GitHub repository, https://github.com/estebangongora/murre-microbiome.
