# Supplementary material for: Gut microbiome is affected by inter-sexual and inter-seasonal variation in diet for thick-billed murres (Uria lomvia)
Source: Sci Rep. 2021 Jan 13;11:1200. doi: 10.1038/s41598-020-80557-x (PMC7806582; doi:10.1038/s41598-020-80557-x)
Supplement: Supplementary file 1 — Supplementary Information. [file 41598_2020_80557_MOESM1_ESM.pdf]

Supplementary Information for Gut microbiome is affected by inter-sexual and inter-seasonal variation in diet for thick-billed murres (*Uria lomvia*)

Esteban Góngora[1]*, Kyle H. Elliott[1], and Lyle Whyte[1]

[1]Department of Natural Resource Sciences, McGill University, 21111 Lakeshore Road, Sainte-Anne-de-Bellevue, Canada, H9X 3V9

*Corresponding author: Esteban Góngora, Department of Natural Resource Sciences, McGill University, 21111 Lakeshore Road, Sainte-Anne-de-Bellevue, Canada, H9X 3V9, Telephone: +(1) 514-398-7891, Email: esteban.gongora@mail.mcgill.ca

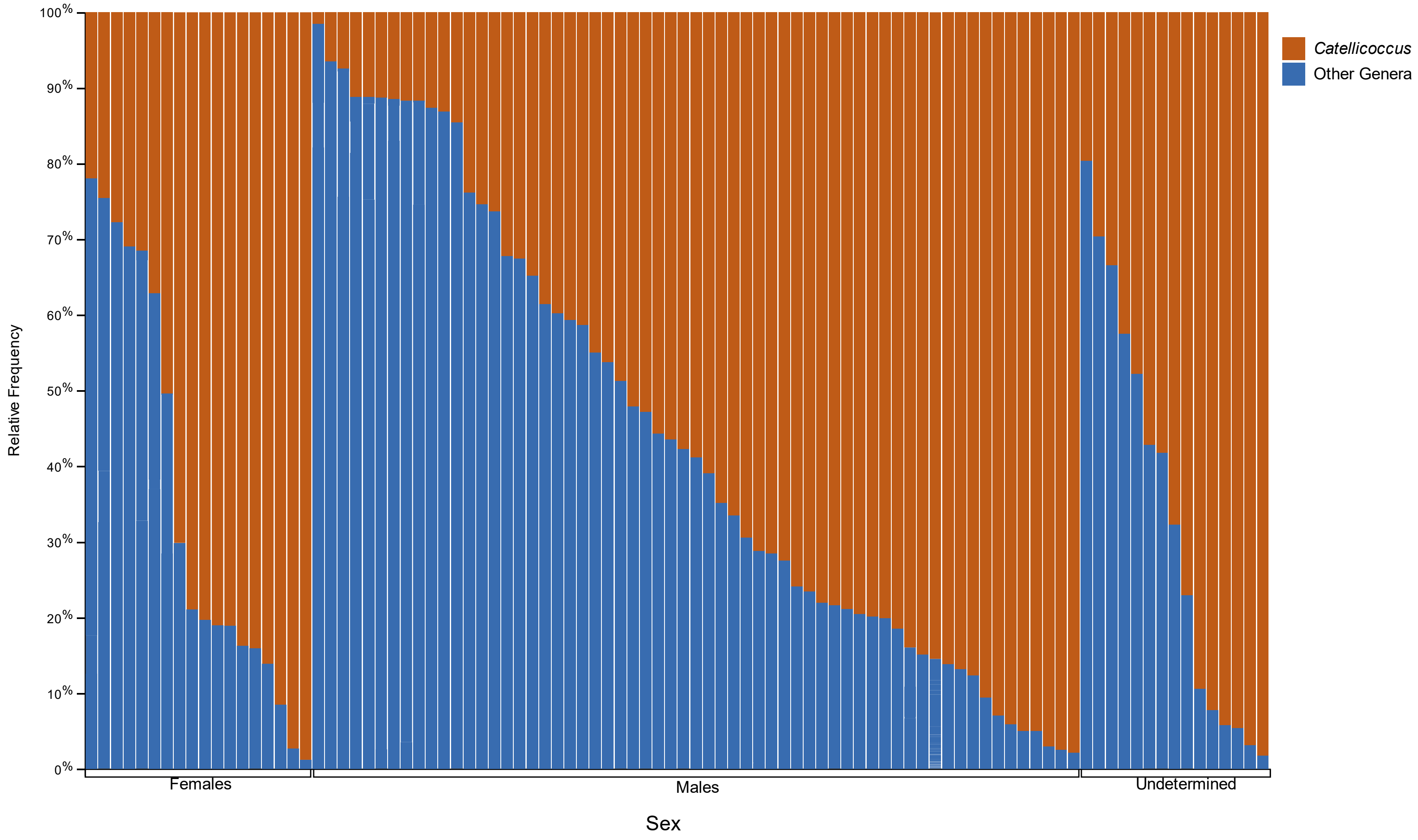

Supplementary Figure 1. Taxonomic classification at the Genus level of the ASVs obtained from murre fecal samples separated by the sex of the sampled birds. A strong influence of bacteria belonging to the genus Catellicoccus, compared to all other genera, can be observed.

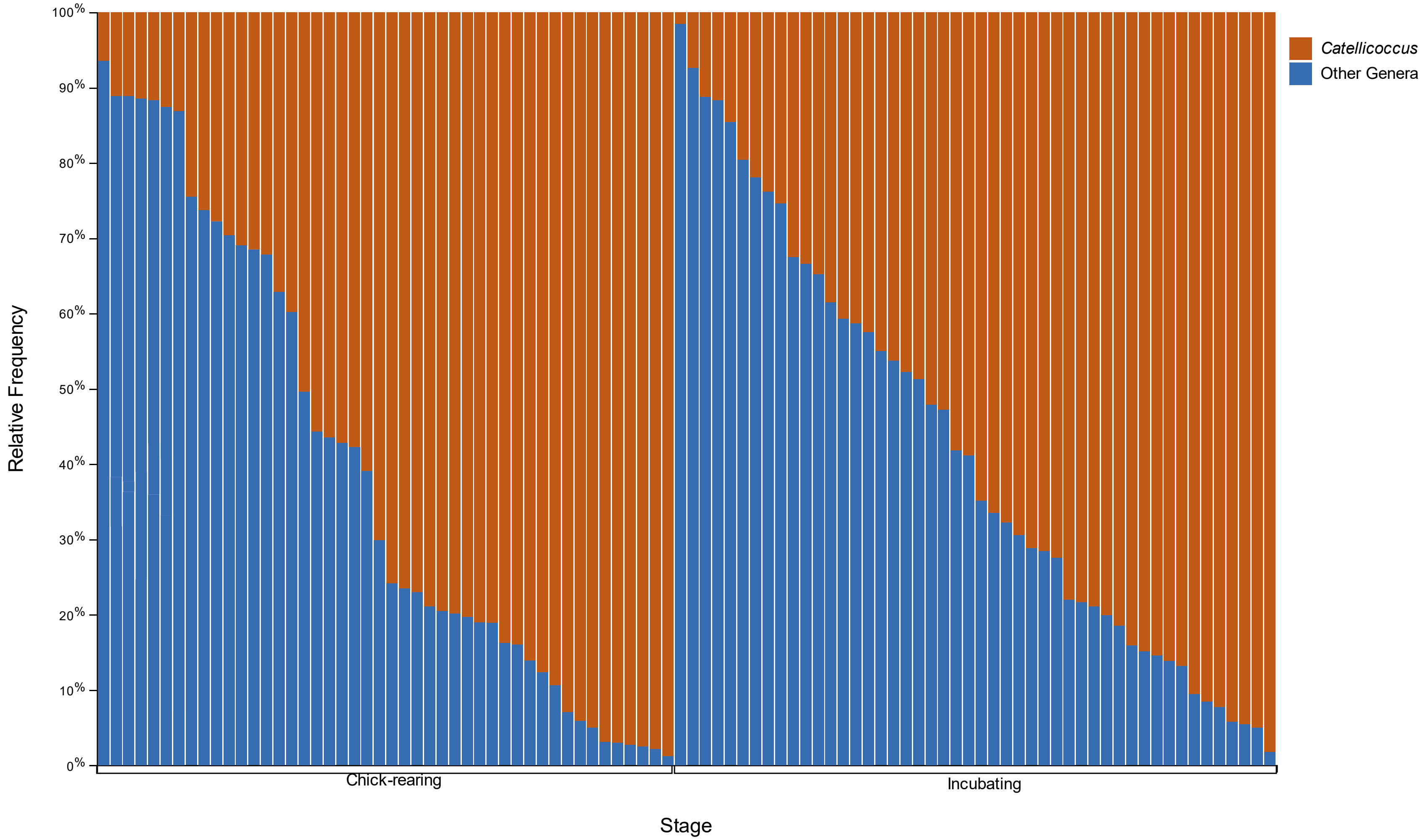

Supplementary Figure 2. Taxonomic classification at the Genus level of the ASVs obtained from murre fecal samples separated by the reproductive stage at which the bird was during sampling. A strong influence of bacteria belonging to the genus Catellicoccus, compared to all other genera, can be observed.

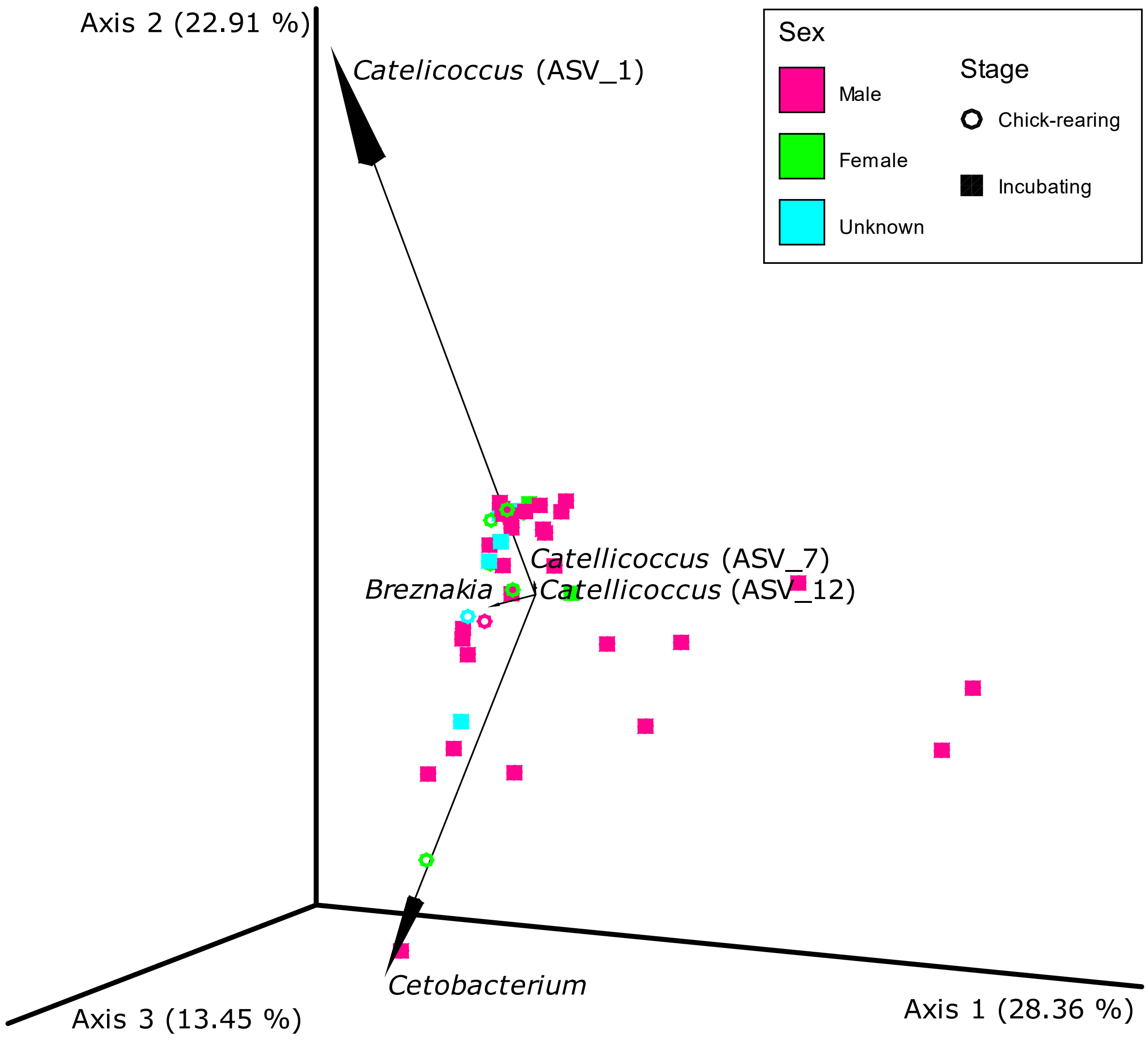

Supplementary Figure 3. Principal Coordinates Analysis (PCoA) plot for the gut community composition using the weighted UniFrac metric with samples identified by sex and reproductive stage. Biplots show the taxonomy of the ASVs with the top 5 effects on the community composition with position of the arrow indicating the direction of the effect.

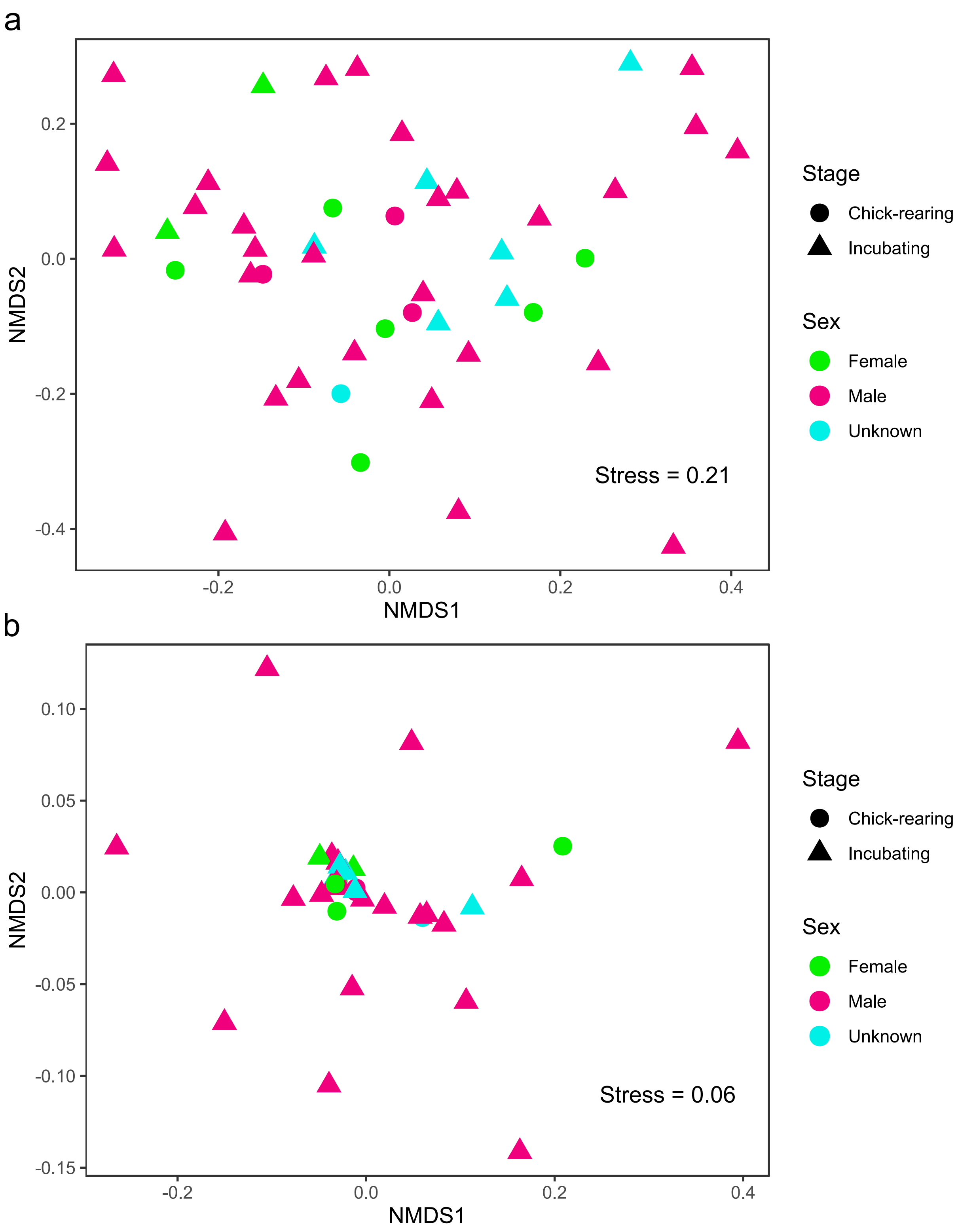

Supplementary Figure 4. Non-metric Multi-dimensional Scaling (NMDS) plots for the gut community composition using the (**a**) unweighted UniFrac and (**b**) weighted UniFrac metrics with samples identified by sex and reproductive stage.

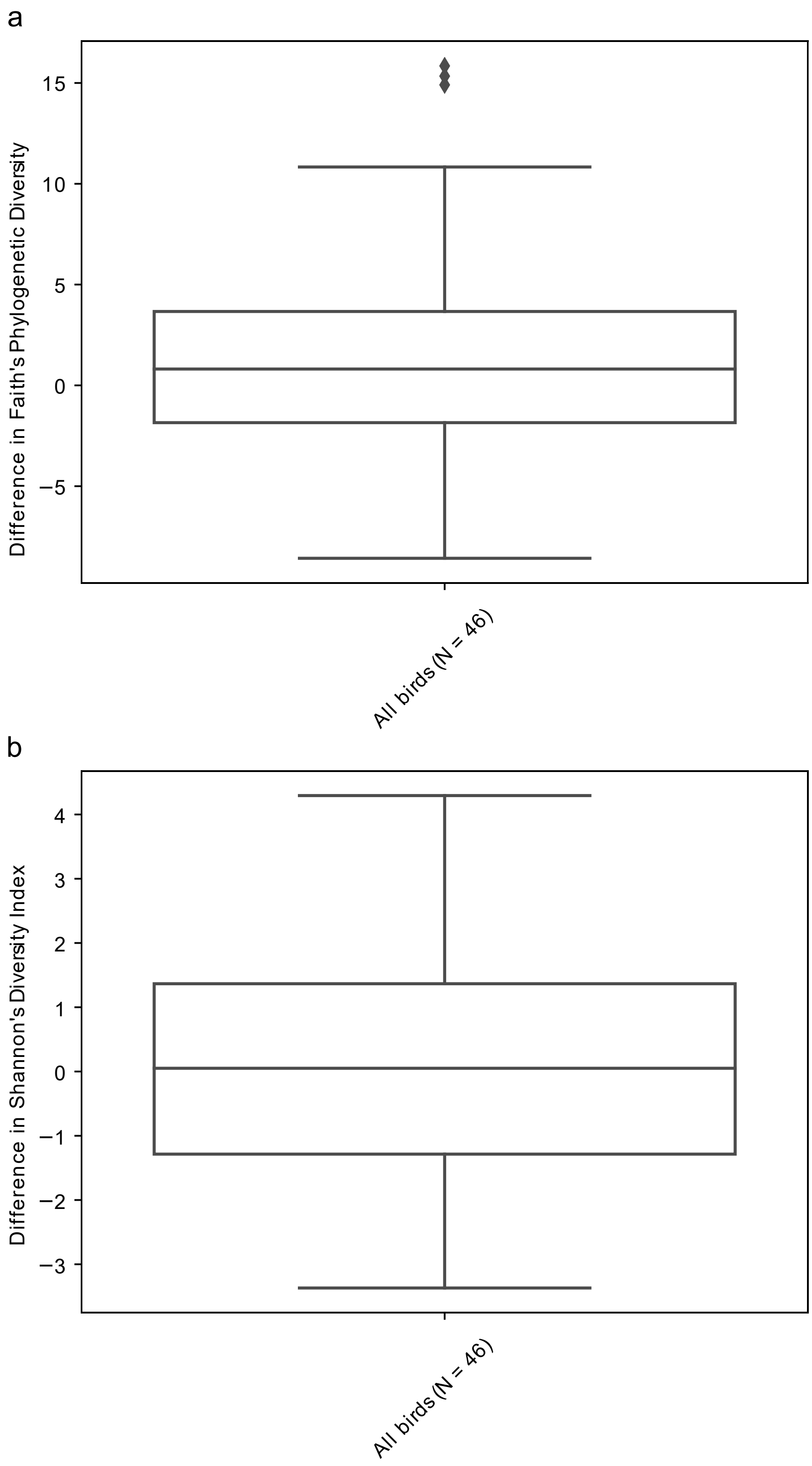

Supplementary Figure 5. Differences in (**a**) Faith's phylogenetic diversity and (**b**) Shannon's Diversity Index between samples from the same bird in the two studied time points.

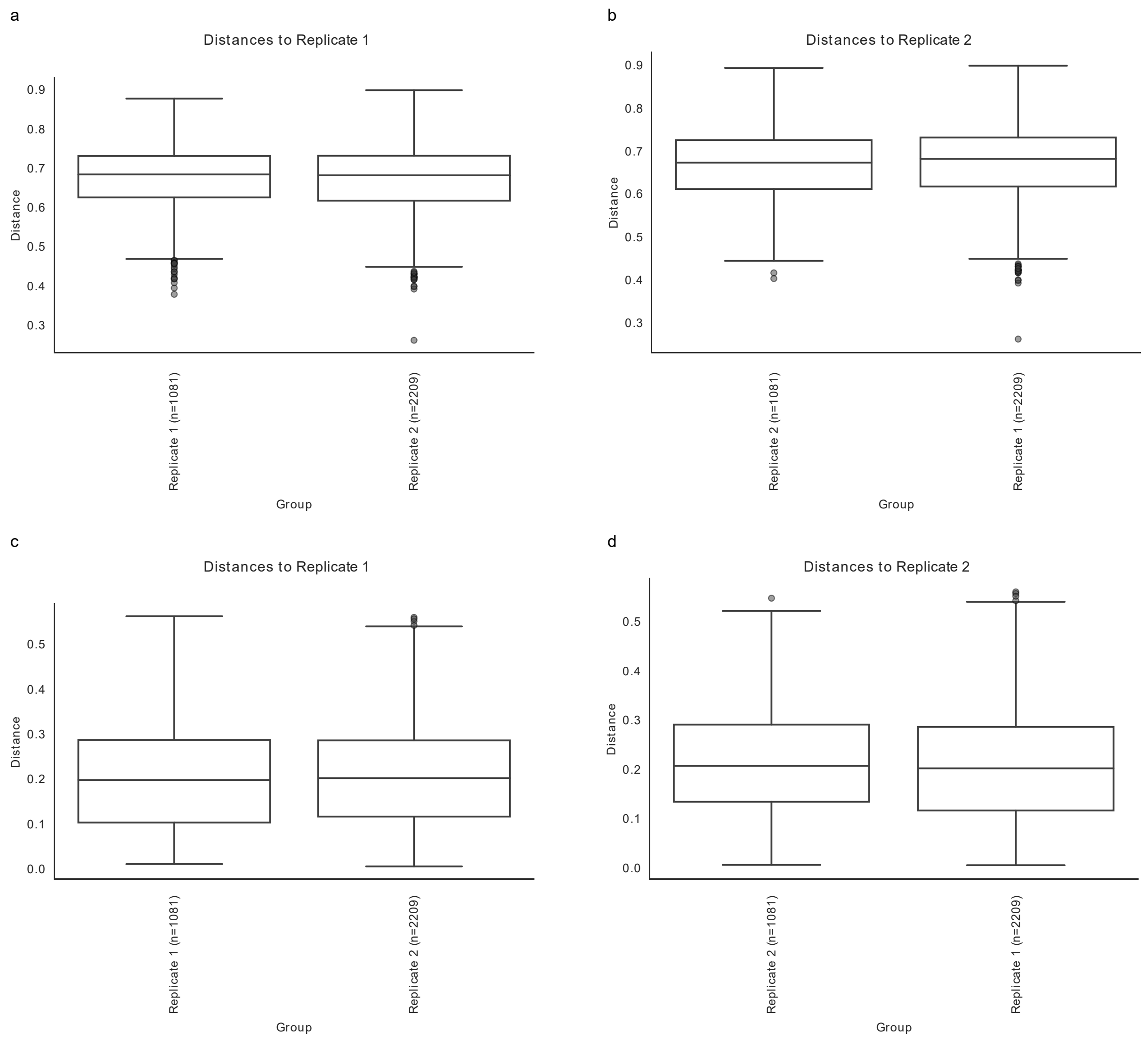

Supplementary Figure 6. Differences in (**a** and **b**) unweighted UniFrac (**c** and **d**) and weighted UniFrac distances between samples from different sampling points or among different samples from the same sampling points showing that there are no detectable differences between sampling points.

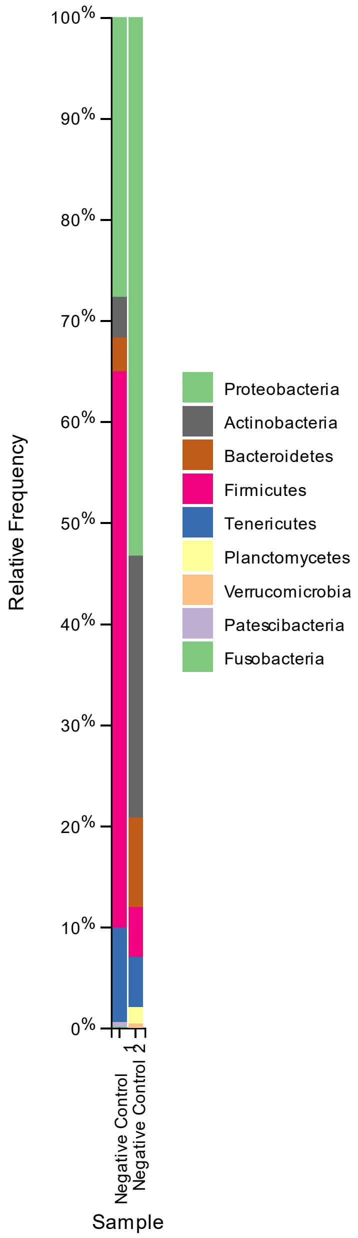

Supplementary Figure 7. Taxonomic classification at the Phylum level of the ASVs detected in the negative controls which were removed from the samples of the present study.

Table S1. DESeq2 results showing differences in ASV abundance between sexes or seasons along with the taxonomy of each of these ASVs.

Row	Category	Comparison	Base Mean	log2 Fold Change	log2 Fold Change SE	Wald Statistic	*p*	p_{adj}
1	Sex	Male vs Female	23.3049525	21.05287543	2.922304317	7.204203652	5.84E-13	1.30E-11
2	Sex	Male vs Female	29.4750752	23.06125581	3.427710075	6.727889844	1.72E-11	2.39E-10
3	Sex	Male vs Female	28.7521648	6.12947426	2.051538158	2.987745676	0.00281	0.023997
4	Sex	Male vs Female	42.2219483	23.55571092	3.678459901	6.40368838	1.52E-10	1.87E-09
5	Sex	Male vs Female	24.7476437	22.81730019	3.678723734	6.202504413	5.56E-10	5.61E-09
6	Sex	Male vs Female	9.70400599	21.50691554	3.094951926	6.949030566	3.68E-12	5.83E-11
7	Sex	Male vs Female	43.7941691	23.53157484	2.824498825	8.331239026	8.00E-17	7.85E-15
8	Sex	Male vs Female	37.2035401	23.38799891	2.91252211	8.030153257	9.74E-16	3.60E-14
9	Sex	Male vs Female	43.5999389	23.60857436	2.952953097	7.994903267	1.30E-15	3.60E-14
10	Sex	Male vs Female	361.036003	6.932628823	1.952405383	3.550814233	0.000384	0.003552
11	Sex	Male vs Female	26.1652012	21.93409943	3.110125012	7.052481601	1.76E-12	3.25E-11
12	Sex	Male vs Female	27.1588966	7.520732346	2.551233157	2.947881234	0.0032	0.025368
13	Sex	Male vs Female	34.2336819	23.26667586	3.678547267	6.324963139	2.53E-10	2.81E-09
14	Sex	Male vs Female	22.3547708	22.67662055	2.744188346	8.263507343	1.41E-16	7.85E-15
15	Sex	Male vs Female	183.334153	-2.402760468	0.861766867	-2.788179217	0.005301	0.039224
16	Stage	Chick-rearing vs Incubating	11.5392506	4.966452558	1.96570865	2.526545608	0.011519	0.04409

17	Stage	Chick-rearing vs Incubating	24.7496158	22.80595425	2.723179708	8.374751833	5.53E-17	7.62E-16
18	Stage	Chick-rearing vs Incubating	71.5661805	8.907157413	3.581328669	2.48710974	0.012879	0.047651
19	Stage	Chick-rearing vs Incubating	21.1338166	22.59696445	2.965206921	7.620704071	2.52E-14	2.80E-13
20	Stage	Chick-rearing vs Incubating	15.3129338	7.25890192	2.792979012	2.598981907	0.00935	0.044049
21	Stage	Chick-rearing vs Incubating	37.8904604	9.159982677	3.58395341	2.555831962	0.010593	0.04409
22	Stage	Chick-rearing vs Incubating	31.7024425	-6.78627885	2.593237118	-2.616914127	0.008873	0.044049
23	Stage	Chick-rearing vs Incubating	11.6481111	6.304012704	2.476473838	2.545559985	0.01091	0.04409
24	Stage	Chick-rearing vs Incubating	221.989994	-4.191451611	1.638425252	-2.558219612	0.010521	0.04409
25	Stage	Chick-rearing vs Incubating	24.7306109	22.75387446	2.721165448	8.361812207	6.18E-17	7.62E-16
26	Stage	Chick-rearing vs Incubating	52.38769	7.761698295	2.094280528	3.706140697	0.00021	0.001795
27	Stage	Chick-rearing vs Incubating	18.1565593	6.764604851	2.342924357	2.887248506	0.003886	0.023943
28	Stage	Chick-rearing vs Incubating	12.5616797	6.405362706	2.470591381	2.592643508	0.009524	0.044049
29	Stage	Chick-rearing vs Incubating	42.7847936	23.57396784	2.411964288	9.773763217	1.46E-22	8.10E-21
30	Stage	Chick-rearing vs Incubating	36.2515828	23.35002833	2.479559442	9.417006883	4.64E-21	1.29E-19
31	Stage	Chick-rearing vs Incubating	40.7390693	23.46120419	2.410614916	9.732456243	2.19E-22	8.11E-21
32	Stage	Chick-rearing vs Incubating	347.811666	6.912170052	1.582616086	4.367559584	1.26E-05	0.000116
33	Stage	Chick-rearing vs Incubating	70.7498483	4.702549123	1.638240608	2.870487461	0.004098	0.023943
34	Stage	Chick-rearing vs Incubating	84.7802262	8.712679829	2.694433995	3.233584436	0.001222	0.007982
35	Stage	Chick-rearing vs Incubating	52.6838393	22.97777326	2.706878263	8.488661485	2.09E-17	3.31E-16
36	Stage	Chick-rearing vs Incubating	25.4571288	7.695024651	2.12316969	3.62430977	0.00029	0.002144

37	Stage	Chick-rearing vs Incubating	151.890594	5.259961427	1.575457161	3.338688958	0.000842	0.00584
38	Stage	Chick-rearing vs Incubating	15.0641958	6.024789981	2.130890349	2.827358049	0.004693	0.026048
39	Stage	Chick-rearing vs Incubating	21.8236184	22.53002968	2.248311411	10.02086702	1.23E-23	1.37E-21
40	Stage	Chick-rearing vs Incubating	24.190142	22.77796955	2.558433531	8.903092174	5.43E-19	1.00E-17
41	Stage	Chick-rearing vs Incubating	525.505464	5.435002816	1.473894565	3.687511267	0.000226	0.001795
42	Stage	Chick-rearing vs Incubating	30.3254601	23.09377285	3.584044836	6.443494406	1.17E-10	1.18E-09
43	Stage	Chick-rearing vs Incubating	21.1219866	21.35791962	2.345590988	9.105560058	8.58E-20	1.91E-18
44	Stage	Chick-rearing vs Incubating	103.085346	5.143652055	1.895459722	2.713669933	0.006654	0.035172
45	Stage	Chick-rearing vs Incubating	98.0437356	1.886452082	0.745532436	2.530342062	0.011395	0.04409

Table S1 (cont.)

Row	Phylum	Class	Order	Family	Genus
1	Bacteroidetes	Bacteroidia	Bacteroidales	Porphyromonadaceae	*Porphyromonas*
2	Fusobacteria	Fusobacteriia	Fusobacteriales	Fusobacteriaceae	*Cetobacterium*
3	Fusobacteria	Fusobacteriia	Fusobacteriales	Fusobacteriaceae	*Cetobacterium*
4	Proteobacteria	Gammaproteobacteria	Pseudomonadales	Moraxellaceae	*Enhydrobacter*
5	Proteobacteria	Gammaproteobacteria	Oceanospirillales	Halomonadaceae	*Halomonas*
6	Actinobacteria	Actinobacteria	Micrococcales	Micrococcaceae	*Micrococcus*
7	Firmicutes	Clostridia	Clostridiales	Family XI	*Tissierella*
8	Firmicutes	Clostridia	Clostridiales	Family XI	*Tissierella*
9	Firmicutes	Clostridia	Clostridiales	Family XI	*Gottschalkia*
10	Firmicutes	Clostridia	Clostridiales	Family XI	*Gottschalkia*
11	Firmicutes	Erysipelotrichia	Erysipelotrichales	Erysipelotrichaceae	NA
12	Firmicutes	Erysipelotrichia	Erysipelotrichales	Erysipelotrichaceae	*Erysipelothrix*
13	Firmicutes	Bacilli	Bacillales	Bacillaceae	*Bacillus*

14	Firmicutes	Bacilli	Bacillales	Bacillaceae	NA
15	Firmicutes	Bacilli	Lactobacillales	Enterococcaceae	*Catellicoccus*
16	Bacteroidetes	Bacteroidia	Bacteroidales	Porphyromonadaceae	*Porphyromonas*
17	Bacteroidetes	Bacteroidia	Bacteroidales	Porphyromonadaceae	*Porphyromonas*
18	Tenericutes	Mollicutes	Mycoplasmatales	Mycoplasmataceae	*Mycoplasma*
19	Fusobacteria	Fusobacteriia	Fusobacteriales	Fusobacteriaceae	*Cetobacterium*
20	Proteobacteria	Gammaproteobacteria	Pseudomonadales	Moraxellaceae	*Acinetobacter*
21	Proteobacteria	Gammaproteobacteria	Pseudomonadales	Moraxellaceae	*Enhydrobacter*
22	Actinobacteria	Actinobacteria	NA	NA	NA
23	Actinobacteria	Actinobacteria	Micrococcales	Micrococcaceae	*Micrococcus*
24	Firmicutes	Clostridia	Clostridiales	Ruminococcaceae	*Fastidiosipila*
25	Chlamydiae	Chlamydiae	Chlamydiales	Chlamydiaceae	*Chlamydia*
26	Firmicutes	Clostridia	Clostridiales	Clostridiaceae 1	*Clostridium sensu stricto 1*
27	Firmicutes	Clostridia	Clostridiales	Clostridiaceae 1	*Clostridium sensu stricto 1*
28	Firmicutes	Clostridia	Clostridiales	Family XI	NA
29	Firmicutes	Clostridia	Clostridiales	Family XI	*Tissierella*
30	Firmicutes	Clostridia	Clostridiales	Family XI	*Tissierella*
31	Firmicutes	Clostridia	Clostridiales	Family XI	*Gottschalkia*
32	Firmicutes	Clostridia	Clostridiales	Family XI	*Gottschalkia*
33	Firmicutes	Clostridia	Clostridiales	Family XI	*Gottschalkia*
34	Firmicutes	Clostridia	Clostridiales	Family XI	*Parvimonas*
35	Firmicutes	Erysipelotrichia	Erysipelotrichales	Erysipelotrichaceae	NA
36	Firmicutes	Erysipelotrichia	Erysipelotrichales	Erysipelotrichaceae	*Erysipelothrix*
37	Firmicutes	Bacilli	Lactobacillales	Carnobacteriaceae	*Atopostipes*
38	Firmicutes	Bacilli	Lactobacillales	Carnobacteriaceae	*Atopostipes*
39	Firmicutes	Bacilli	Lactobacillales	Carnobacteriaceae	NA
40	Firmicutes	Bacilli	Lactobacillales	Carnobacteriaceae	*Atopostipes*
41	Firmicutes	Bacilli	Lactobacillales	Lactobacillaceae	*Lactobacillus*
42	Firmicutes	Bacilli	Bacillales	Bacillaceae	*Bacillus*

43	Firmicutes	Bacilli	Bacillales	Bacillaceae	NA
44	Firmicutes	Bacilli	Bacillales	Bacillaceae	NA
45	Firmicutes	Bacilli	Lactobacillales	Enterococcaceae	*Catellicoccus*

Table S2. Stable isotope ratio, sexing, and sample collection data of thick-billed murre samples collected at Coats Island, Canada.

SampleID	BirdID	Date	Sample	Color	$\delta^{13}C$	%C	$\delta^{15}N$	%N	$\delta^{34}S$	%S	Sex	Status
EGT1	99676429	10/07/2017	1	W	N/A	N/A	N/A	N/A	N/A	N/A	N/A	Incubating
EGT100	99658331	28/07/2017	1	YW	-20.75	42.4	15.52	13.17	18.04	0.66	N/A	Chick-rearing
EGT101	117639797	28/07/2017	1	W	N/A	N/A	N/A	N/A	N/A	N/A	F	Chick-rearing
EGT102	99679694	28/07/2017	1	RW	-20.34	48.2	15.27	15.4	19.49	0.69	F	Chick-rearing
EGT103	99656411	28/07/2017	1	Y	-20.66	48.2	14.11	15	17.03	0.72	M	Chick-rearing
EGT104	99680764	28/07/2017	1	W	N/A	N/A	N/A	N/A	N/A	N/A	M	Chick-rearing
EGT105	118606010	28/07/2017	1	W	-20.43	49.9	15.59	15.3	19.57	0.64	F	Chick-rearing
EGT106	99653374	28/07/2017	1	RW	-21.14	48.2	15.33	13.89	16.45	0.74	F	Chick-rearing
EGT107	99653374	01/08/2017	2	R	N/A	N/A	N/A	N/A	N/A	N/A	F	Chick-rearing
EGT108	99680540	01/08/2017	2	RW	N/A	N/A	N/A	N/A	N/A	N/A	F	Chick-rearing
EGT109	99656411	02/08/2017	2	W	N/A	N/A	N/A	N/A	N/A	N/A	M	Chick-rearing
EGT11	99670720	06/07/2017	2	Y	-20.31	48.8	13.83	15.2	18.69	0.61	M	Incubating
EGT110	117639823	01/08/2017	2	RW	N/A	N/A	N/A	N/A	N/A	N/A	F	Chick-rearing
EGT111	99679694	01/08/2017	2	Y	N/A	N/A	N/A	N/A	N/A	N/A	F	Chick-rearing
EGT112	99658331	01/08/2017	2	RW	N/A	N/A	N/A	N/A	N/A	N/A	N/A	Chick-rearing
EGT113	99680764	01/08/2017	2	W	N/A	N/A	N/A	N/A	N/A	N/A	M	Chick-rearing
EGT114	118606010	01/08/2017	2	YG	N/A	N/A	N/A	N/A	N/A	N/A	F	Chick-rearing
EGT115	99676921	01/08/2017	2	R	N/A	N/A	N/A	N/A	N/A	N/A	F	Chick-rearing
EGT116	117639797	01/08/2017	2	Y	N/A	N/A	N/A	N/A	N/A	N/A	F	Chick-rearing
EGT12	99686990	07/07/2017	2	Y	-20.44	49.7	14.67	15.3	19.57	0.82	M	Incubating
EGT13	99667063	09/07/2017	2	R	-20.58	48.2	14.29	15.1	17.24	0.73	M	Incubating
EGT14	99686180	09/07/2017	2	Y	-20.67	47.8	14.39	14.86	19.61	0.71	M	Incubating
EGT17	99676429	12/07/2017	2	Y	N/A	N/A	N/A	N/A	N/A	N/A	N/A	Incubating
EGT18	117639776	12/07/2017	2	W	-20.97	50.1	14.71	14.52	19.28	0.74	N/A	Incubating
EGT19	99676337	06/07/2017	1	Y	N/A	N/A	N/A	N/A	N/A	N/A	F	Incubating
EGT20	99676338	06/07/2017	1	R	N/A	N/A	N/A	N/A	N/A	N/A	M	Incubating
EGT21	99696862	12/07/2017	2	Y	N/A	N/A	N/A	N/A	N/A	N/A	N/A	Incubating
EGT22	99667218	10/07/2017	1	Y	N/A	N/A	N/A	N/A	N/A	N/A	M	Incubating

EGT23	117639773	10/07/2017	1	Y	N/A	N/A	N/A	N/A	N/A	N/A	N/A	Incubating
EGT24	99676338	10/07/2017	2	R	-20.42	49	14.14	15.5	18.59	0.72	M	Incubating
EGT25	117639773	12/07/2017	2	W	-20.23	50.1	15.12	15.5	18.99	0.73	N/A	Incubating
EGT37	99697231	17/07/2017	2	W	N/A	N/A	N/A	N/A	N/A	N/A	N/A	Chick-rearing
EGT38	99683053	17/07/2017	2	Y	N/A	N/A	N/A	N/A	N/A	N/A	M	Chick-rearing
EGT39	99676555	17/07/2017	2	W	N/A	N/A	N/A	N/A	N/A	N/A	M	Chick-rearing
EGT40	99699833	17/07/2017	2	YG	N/A	N/A	N/A	N/A	N/A	N/A	M	Chick-rearing
EGT41	99687257	17/07/2017	2	W	N/A	N/A	N/A	N/A	N/A	N/A	M	Chick-rearing
EGT42	99600376	15/07/2017	1	W	-21.07	46.7	14.29	13.97	18.25	0.47	M	Incubating
EGT43	118600092	17/07/2017	2	YW	N/A	N/A	N/A	N/A	N/A	N/A	M	Chick-rearing
EGT44	99689936	17/07/2017	2	YW	N/A	N/A	N/A	N/A	N/A	N/A	M	Chick-rearing
EGT45	99659671	14/07/2017	1	Y	-20.47	60	14.19	18	18	0.44	M	Incubating
EGT46	99664156	17/07/2017	2	YG	N/A	N/A	N/A	N/A	N/A	N/A	N/A	Chick-rearing
EGT48	99687257	14/07/2017	1	W	-20.62	35.4	14.15	10.6	17.77	0.81	M	Incubating
EGT5	117639776	10/07/2017	1	W	N/A	N/A	N/A	N/A	N/A	N/A	N/A	Incubating
EGT50	118607286	17/07/2017	2	YG	N/A	N/A	N/A	N/A	N/A	N/A	M	Chick-rearing
EGT52	99683053	14/07/2017	1	YG	-20.51	13.52	13.76	3.97	17.56	0.82	M	Incubating
EGT53	99684228	15/07/2017	1	Y	-20.6	56.7	14.52	16.8	18.06	0.46	M	Incubating
EGT55	118600400	17/07/2017	2	Y	N/A	N/A	N/A	N/A	N/A	N/A	N/A	Chick-rearing
EGT56	99689234	17/07/2017	2	Y	N/A	N/A	N/A	N/A	N/A	N/A	M	Chick-rearing
EGT57	99667218	13/07/2017	2	W	-20.35	48.3	14.86	15.1	17.91	0.8	M	Incubating
EGT58	99659671	17/07/2017	2	Y	N/A	N/A	N/A	N/A	N/A	N/A	M	Chick-rearing
EGT59	99683052	17/07/2017	2	Y	N/A	N/A	N/A	N/A	N/A	N/A	M	Chick-rearing
EGT6	99670720	17/07/2017	2	RW	N/A	N/A	N/A	N/A	N/A	N/A	M	Chick-rearing
EGT60	99680514	17/07/2017	2	Y	N/A	N/A	N/A	N/A	N/A	N/A	F	Chick-rearing
EGT61	99689147	17/07/2017	2	Y	N/A	N/A	N/A	N/A	N/A	N/A	M	Chick-rearing
EGT62	99684228	17/07/2017	2	W	N/A	N/A	N/A	N/A	N/A	N/A	M	Chick-rearing
EGT63	118607294	17/07/2017	2	Y	N/A	N/A	N/A	N/A	N/A	N/A	M	Chick-rearing
EGT64	99670801	17/07/2017	2	Y	N/A	N/A	N/A	N/A	N/A	N/A	M	Chick-rearing
EGT65	99689936	15/07/2017	1	RW	-20.45	43.6	14.31	13.14	18.08	0.45	M	Incubating
EGT66	99699835	17/07/2017	2	W	N/A	N/A	N/A	N/A	N/A	N/A	M	Chick-rearing

EGT67	99689234	14/07/2017	1	W	-20.51	51.2	14.31	15.2	17.9	0.39	M	Incubating
EGT68	118600092	14/07/2017	1	Y	-20.83	53.4	14.3	15.8	17.36	0.82	M	Incubating
EGT7	99686990	03/07/2017	1	R	N/A	N/A	N/A	N/A	N/A	N/A	M	Incubating
EGT70	118607294	14/07/2017	1	Y	-20.57	42.6	14.81	12.67	18.17	0.42	M	Incubating
EGT71	118607286	14/07/2017	1	R	-20.38	39.3	14.96	11.58	18.02	0.38	M	Incubating
EGT72	118607282	14/07/2017	1	W	-20.55	36.3	14.02	10.74	17.39	0.82	M	Incubating
EGT73	99665084	14/07/2017	1	Y	-21.22	42.7	14	12.64	17.93	0.39	M	Incubating
EGT74	118600400	14/07/2017	1	W	-20.14	55.4	15.07	16.3	17.63	0.8	N/A	Incubating
EGT75	99689147	14/07/2017	1	YW	-20.29	46.8	14.27	14.05	18	0.42	M	Incubating
EGT76	99676614	14/07/2017	1	Y	-20.41	52.4	14.32	15.7	18.14	0.43	M	Incubating
EGT77	99676337	13/07/2017	2	Y	-20.39	49	15.07	15.3	18.74	0.72	F	Incubating
EGT78	118607282	17/07/2017	2	W	N/A	N/A	N/A	N/A	N/A	N/A	M	Chick-rearing
EGT79	99683052	14/07/2017	1	Y	-21.12	43.3	14.39	12.92	17.48	0.81	M	Incubating
EGT8	99667063	03/07/2017	1	R	N/A	N/A	N/A	N/A	N/A	N/A	M	Incubating
EGT80	99680748	14/07/2017	1	W	-20.56	51	14.68	15.1	18.35	0.46	M	Incubating
EGT81	99699837	15/07/2017	1	YG	-20.43	48.2	14.46	14.44	17.85	0.4	M	Incubating
EGT82	99699832	15/07/2017	1	W	N/A	N/A	N/A	N/A	N/A	N/A	M	Incubating
EGT83	99699835	15/07/2017	1	W	-21.18	42.1	14.18	12.57	18.07	0.51	M	Incubating
EGT84	99600376	17/07/2017	2	R	N/A	N/A	N/A	N/A	N/A	N/A	M	Chick-rearing
EGT85	99699833	15/07/2017	1	Y	-20.31	59.8	14.89	18	18.39	0.52	M	Incubating
EGT86	99670801	15/07/2017	1	Y	-20.39	41.2	14.2	12.33	18.21	0.47	M	Incubating
EGT87	99665084	17/07/2017	2	W	N/A	N/A	N/A	N/A	N/A	N/A	M	Chick-rearing
EGT88	118607287	17/07/2017	2	W	N/A	N/A	N/A	N/A	N/A	N/A	M	Chick-rearing
EGT89	99680514	14/07/2017	1	Y	-20.51	58.4	14.82	17.2	17.53	0.79	F	Incubating
EGT9	99686180	03/07/2017	1	Y	N/A	N/A	N/A	N/A	N/A	N/A	M	Incubating
EGT90	99664156	14/07/2017	1	Y	-20.37	34.5	14.44	10.2	17.91	0.85	N/A	Incubating
EGT91	99699837	17/07/2017	2	YG	N/A	N/A	N/A	N/A	N/A	N/A	M	Chick-rearing
EGT92	118607287	14/07/2017	1	YW	-20.36	50.1	14.74	15.1	17.98	0.41	M	Incubating
EGT93	99676555	14/07/2017	1	Y	-20.37	46.8	14.5	13.91	17.85	0.83	M	Incubating
EGT94	99699832	17/07/2017	2	Y	-20.38	31.7	14.43	9.33	18.23	0.51	M	Chick-rearing
EGT95	99676614	17/07/2017	2	YW	N/A	N/A	N/A	N/A	N/A	N/A	M	Chick-rearing

EGT96	99697231	15/07/2017	1	RW	N/A	N/A	N/A	N/A	N/A	N/A	N/A	Incubating
EGT97	99676921	28/07/2017	1	R	-20.35	49	16.09	15.4	19.44	0.71	F	Chick-rearing
EGT98	117639823	28/07/2017	1	W	-20.35	48.7	15.45	14.9	19.68	0.65	F	Chick-rearing
EGT99	99680540	28/07/2017	1	W	-20.76	48.7	15.63	14.95	19.99	0.72	F	Chick-rearing
